# Neglect and Neurodevelopment: A Narrative Review Understanding the Link Between Child Neglect and Executive Function Deficits

**DOI:** 10.3390/biomedicines13071565

**Published:** 2025-06-26

**Authors:** Silvia Herrero-Roldán, Alexandra Martín-Rodríguez

**Affiliations:** 1 Faculty of Health Sciences, UNIE University, 28015 Madrid, Spain; silvia.herrero@universidadunie.com; 2 Faculty of Education Sciences, UNIE University, 28015 Madrid, Spain; 3 Faculty of Medicine, Health and Sports, Universidad Europea de Madrid, Villaviciosa de Odón, 28670 Madrid, Spain

**Keywords:** child neglect, executive functions, neurodevelopment, prefrontal cortex, HPA axis, epigenetics

## Abstract

**Background**: Childhood neglect is a pervasive yet often overlooked form of maltreatment that exerts profound and lasting effects on neurodevelopment. Unlike other types of abuse, neglect is characterized by the absence of essential stimuli and caregiving, which are critical for normal brain maturation, particularly in regions involved in executive function. **Objective**: This narrative review aims to critically explore the neurobiological mechanisms through which early-life neglect impairs the development of executive functions. Special emphasis is placed on alterations in brain structure and function, dysregulation of the hypothalamic–pituitary–adrenal (HPA) axis, and emerging epigenetic evidence. **Methods**: A comprehensive literature search (170 articles) was conducted across PubMed, Scopus, Web of Science, and PsycINFO, including studies published between 1 January 2000 to 31 March 2025. Relevant empirical and review articles were selected based on methodological rigor, relevance to executive functioning, and focus on child neglect. **Results**: Evidence reveals that neglect disrupts key neural circuits, particularly those involving the prefrontal cortex and amygdala, leading to deficits in attention, working memory, impulse control, and cognitive flexibility. Chronic stress associated with neglect also induces HPA axis dysregulation and elevated cortisol levels, which further compromise neural plasticity. Additionally, epigenetic modifications appear to mediate long-term cognitive and emotional consequences. **Conclusions**: Childhood neglect represents a distinct and critical risk factor for executive dysfunction. Understanding the neurodevelopmental consequences of neglect is essential for developing targeted prevention strategies and therapeutic interventions aimed at supporting cognitive resilience in affected populations.

## 1. Introduction: Prevalence and Conceptualization of Child Neglect

Contrary to what one might assume, neglect is the most common and, in many cases, the most severe form of child maltreatment, surpassing even physical and sexual abuse in prevalence. This trend is consistently observed worldwide. For example, in Spain, approximately 50% of reported child maltreatment cases are related to neglect [1,2], a percentage that rises to 51.2% in Mexico [1] and reaches a concerning 75.3% in the United States [3]. However, neglect continues to be the most difficult form of child maltreatment to detect since, unlike other forms, it does not involve a proactive act toward the child but rather constitutes maltreatment by omission [4]. As a result, only the most extreme and least representative cases of this type are often identified.

One of the main challenges in addressing this form of child maltreatment is the absence of a unified and objective definition. This lack of consensus has led to multiple interpretations by different researchers, which in turn has resulted in misdiagnoses [5]. As an operational approach, Child Protective Services define neglect as a form of maltreatment in which caregivers or responsible figures consistently fail to meet the basic needs of the children under their care (physical, medical, cognitive, emotional). They also emphasize that such omission is not a result of or cannot be explained by structural conditions such as poverty or a lack of awareness of these needs [6,7]. This conceptualization has received the most support from experts in the field [8,9].

Despite the age of the definition, the categorization from the Child Maltreatment Classification System proposed by Barnett et al. [10] continues to be used, distinguishing four main subtypes of neglect:

- Physical neglect: Refers to the failure to provide essential elements for the child’s survival and development, such as adequate nutrition, appropriate clothing for the weather, personal hygiene, safe living conditions, and proper medical care.
- Emotional neglect: Involves the absence of emotional responsiveness from the caregiver, as well as the failure to attend to the child’s emotional needs, such as not responding to an infant’s cry.
- Lack of supervision: This subtype refers to the absence of appropriate monitoring by responsible adults, which exposes the child to physical, emotional, or psychological risks. It is evident, for instance, when a child is left alone for extended or age-inappropriate periods.
- Educational neglect: Refers to the failure to provide cognitive stimulation and access to basic educational opportunities. This form of child neglect impairs the child’s intellectual, emotional, and social development.

A growing body of evidence suggests that these different subtypes of neglect may exert distinct neurodevelopmental effects, though many existing studies fail to disaggregate these exposures. For instance, physical neglect—which includes malnutrition and lack of medical care—has been particularly associated with alterations in white matter microstructure, likely due to impaired myelination during critical growth windows. In contrast, emotional neglect, such as the absence of caregiver responsiveness or emotional attunement, is more often linked to limbic system dysregulation, including heightened amygdala reactivity and disrupted prefrontal–limbic connectivity [11]. Evidence from both animal and human studies supports these distinctions. Animal studies by Weaver et al. (2004) provided foundational insights into how early caregiving impacts epigenetic regulation of stress-response genes, while Klengel et al. (2013) confirmed these findings in human cohorts exposed to childhood trauma [12,13].

More recent neuroimaging studies have clarified the structural and functional impact of neglect. For instance, Doretto et al. found differential hippocampal volume loss between institutionalized and family-neglected children [14], and Sheridan et al. demonstrated prefrontal cortical thinning in early-deprived samples [15]. Likewise, it has been reported functional alterations in executive networks, and Rosero-Pahi et al. highlighted the timing-specific sensitivity of prefrontal–limbic circuits to neglect exposure [16]. Functional alterations in executive networks have also been documented, underscoring how neglect may compromise higher-order cognitive functions.

Despite these promising findings, few empirical studies isolate specific neglect subtypes, making it difficult to draw firm conclusions about their differential effects. Therefore, we underscore the urgent need for future research to adopt subgroup-sensitive designs, explicitly report neglect categories in samples, and examine how overlapping forms of adversity interact to influence neurodevelopmental outcomes.

For all these reasons, child neglect remains a complex and underestimated phenomenon, despite its high global prevalence. Its passive and often invisible nature makes detection particularly challenging. Clarifying its multiple subtypes—physical, emotional, supervisory, and educational—is essential to improve identification, guide research, and develop effective prevention and intervention strategies for at-risk children.

### 1.1. Research Methodology

This narrative review was conducted following established standards for critical literature synthesis in neuroscience and child psychology. The aim was to integrate empirical findings regarding the impact of childhood neglect on executive function development, with particular attention to neurobiological mechanisms.

A total of 170 articles were reviewed following a comprehensive literature search conducted across four major electronic databases: PubMed, Scopus, Web of Science, and PsycINFO. The search included peer-reviewed articles published between 1 January 2000 to 31 March 2025. We used the following Boolean search strings (adapted to the syntax of each database):

- “child neglect” AND “executive function”
- “child neglect” AND “prefrontal cortex”
- “child neglect” AND “HPA axis”
- “child neglect” AND “brain development”
- “child neglect” AND “epigenetics”
- “early adversity” AND “executive function”
- “maltreatment” AND “executive dysfunction”
- “institutionalization” AND “neurodevelopment”

These terms were adapted as necessary to fit each database’s indexing system. The objective was not to exhaustively capture all available studies, as in a systematic review, but rather to identify high-quality and conceptually relevant publications to build an integrative narrative synthesis. Approximately 1200 records were initially identified. Titles and abstracts were reviewed to assess relevance, and a subset of articles was selected for full-text analysis based on their focus on child neglect (distinct from other maltreatment forms) and direct reference to executive function, brain structure, or stress-response mechanisms. In total, 170 empirical and theoretical studies were selected to inform this review. Priority was given to peer-reviewed articles in Q1 journals from neuroscience, psychiatry, developmental psychology, and epigenetics. Studies that did not clearly differentiate neglect from other adverse experiences or lacked relevance to neurodevelopmental outcomes were excluded. Throughout the process, attention was paid to achieving thematic saturation rather than exhaustive coverage, as is consistent with the aims of narrative synthesis.

#### 1.1.1. Inclusion Criteria

(i). Peer-reviewed articles published in English or Spanish;
(ii). Studies involving human subjects or validated animal models;
(iii). Clear distinction between child neglect and other forms of maltreatment;
(iv). Explicit focus on executive functions, brain structure, or neuroendocrine and epigenetic mechanisms.

#### 1.1.2. Exclusion Criteria

(i). Studies focusing exclusively on physical or sexual abuse;
(ii). Reviews or meta-analyses without primary empirical data (unless theoretically pivotal);
(iii). Articles with significant methodological limitations or lacking clear outcome measures.

A multidisciplinary lens was used to analyze selected studies, integrating data from neuroscience, psychology, endocrinology, and developmental psychopathology. This approach enabled a comprehensive understanding of how early neglect biologically embeds risk for cognitive and emotional dysfunction. However, it was also acknowledged potential limitations related to study heterogeneity and reporting clarity. In some cases, studies included mixed populations of children who experienced institutional neglect, familial neglect, or other forms of maltreatment, without fully disaggregating the findings. In particular, some reported outcomes from cohorts with histories of multiple forms of adversity, including abuse and household dysfunction. These studies were included only when neglect-specific data were either identified as the main exposure or could be inferred through subgroup analyses or operational definitions provided by the authors. This selective approach aimed to maximize relevance while acknowledging the complexity and overlap among maltreatment subtypes in the empirical literature.

Thus, the primary objective of this review is to critically examine how early-life neglect influences the development of executive functions through neurobiological mechanisms. By synthesizing findings from diverse research domains, this review aims to provide an integrative framework that highlights the structural, functional, and regulatory brain alterations associated with neglect, emphasizing their implications for cognitive development and mental health across the lifespan

## 2. Impact of Childhood Neglect on Brain Development

In this context, the World Health Organization states that individuals who have experienced or are currently experiencing maltreatment are exposed to elevated levels of chronic stress, a phenomenon closely linked to disruptions in early brain development [17]. It is well established that sustained stress over time leads to the release of maladaptive levels of cortisol, which also has harmful consequences for brain development [18].

Thus, there is a substantial body of scientific literature documenting the relationship between child maltreatment—including neglect—and alterations in brain structure. In particular, brain regions such as the corpus callosum, hippocampus, amygdala, and prefrontal cortex have been shown to be especially vulnerable to adverse influences during early developmental stages [19]. Evidence has shown that maltreatment is associated with a reduction in the volume of the hippocampus and amygdala [14,20,21], as well as in hippocampal white and gray matter [22,23,24]. Moreover, several studies have reported significant reductions in the corpus callosum, with volume decreases of up to 17% [25] and up to an 8% reduction in intracranial volume in individuals who have suffered maltreatment compared to control subjects. These alterations are particularly influenced by the age of onset and duration of the abuse [26]. In this sense, another important aspect is the differential effect observed depending on the developmental stage during which the abuse occurs. In this regard, it has been reported that the volume of the amygdala and hippocampus is especially sensitive during preadolescence and adolescence [27]. This is not surprising considering that the brain does not fully develop until adolescence, as is the case with the prefrontal cortex, a late-developing brain structure highly susceptible to the negative effects of early stress, which does not reach maturity until around age 16 [28]. Consequently, these critical developmental periods appear to render the brain particularly vulnerable to early adverse experiences, leading to structural alterations.

In addition to the structural changes mentioned, child maltreatment and neglect have also been associated with functional alterations in the aforementioned brain regions. Several studies have documented increased activation of these brain areas, particularly in response to threatening stimuli. For example, various studies have found that the amygdala in individuals who have suffered neglect exhibits maladaptive activation [29,30] indicating an exaggerated and potentially poorly adapted response to threat-related cues [31,32].

Given the evidence presented in the literature, it can be concluded that child maltreatment, in any of its forms, generates elevated levels of chronic stress, which negatively impact brain development—particularly during early stages of life—and undoubtedly result in behavioral alterations [33].

### 2.1. Neurofunctional Signatures: Altered Prefrontal–Amygdala Connectivity

The intricate interplay between the prefrontal cortex (PFC) and the amygdala is fundamental to executive functions (EF) and emotional regulation [34]. Disruptions in this circuitry, particularly due to early-life adversities such as child neglect, can lead to significant deficits in these domains. The PFC is pivotal in cognitive control and EF, encompassing processes like attention regulation, inhibitory control, and working memory. It achieves these functions through extensive connections with various brain regions, notably the amygdala, which is central to processing emotional stimuli and generating affective responses. The dynamic interaction between the PFC and amygdala facilitates the modulation of emotional reactions, ensuring appropriate behavioral responses in social contexts [34,35].

Regarding the impact of child neglect on prefrontal–amygdala connectivity, it is known that the exposure to neglect during critical developmental periods can adversely affect the maturation and functionality of the PFC–amygdala circuitry [36]. Such early-life stressors are associated with reduced functional connectivity; some studies such as Cheng’s et al. indicate that individuals with histories of neglect exhibit diminished connectivity between the medial PFC and the amygdala, impairing the PFC’s regulatory influence over amygdala-driven emotional responses [37]. In this regard, the hyperactivity of the amygdala increases its activation in response to emotional stimuli. This fact heightens sensitivity to potential threats, and it is related to a propensity for anxiety-related behaviors [38]. Also, Roda’s study showed evidence of decreased activation in the PFC, particularly in regions responsible for top-down regulation of emotions, leading to challenges in executive control and decision-making processes [39].

### 2.2. Neurobiological Pathways

Childhood neglect exerts profound effects on neurodevelopment. These adverse outcomes are mediated through several neurobiological mechanisms, notably the dysregulation of the hypothalamic–pituitary–adrenal (HPA) axis, chronic elevation of cortisol levels, and impaired synaptic pruning during critical developmental periods [40,41]. These biological disruptions offer insight into how early environmental deprivation becomes biologically embedded in the developing brain.

Central to this framework is the HPA axis, culminating in the release of cortisol, a glucocorticoid hormone that mobilizes energy and modulates various physiological processes during stress [42,43]. In the context of childhood neglect, the absence of responsive caregiving can lead to persistent activation of the HPA axis [44]. This chronic stimulation results in sustained elevated cortisol levels, which have neurotoxic effects on brain regions integral to executive function, such as the PFC and hippocampus [45,46]. Research indicates that children who have experienced maltreatment exhibit altered cortisol reactivity, suggesting a disruption in the normative functioning of the HPA axis. Such dysregulation is associated with difficulties in attention, impulse control, and working memory, core components of executive functioning [47].

Regarding synaptic pruning, it is a critical neurodevelopmental process wherein excess neuronal connections are eliminated to enhance the efficiency of neural networks [48]. This process is experience-dependent and is particularly active during early childhood and adolescence. Neglect during these sensitive periods deprives the child of necessary environmental stimuli, potentially leading to aberrant synaptic pruning [49]. For instance, insufficient pruning in certain areas may result in hyperconnectivity, while excessive pruning in others can lead to reduced connectivity. Both scenarios disrupt the balance required for optimal neural circuit function [50,51]. Also, studies have shown that early maltreatment can alter synaptic density and organization, particularly in the PFC, thereby impairing cognitive processes reliant on these circuits [52]. Thus, and according to Leroux et al., mental disorders tend to be both more prevalent and more severe among individuals who have experienced child abuse. However, it is emphasized that further research is needed to clarify the extent to which these HPA axis alterations are directly associated with the development of mental disorders in this population [53]. Concretely, executive functions such as planning, decision-making, and inhibitory control may be particularly vulnerable in these contexts. It is also known that damage or developmental delays in this region manifest as difficulties in regulating emotions, increased impulsivity, and challenges in adaptive problem-solving. Furthermore, the hippocampus, essential for memory formation and contextualizing experiences, is also adversely affected, leading to deficits in learning and memory consolidation [54,55].

### 2.3. Neuroimaging Advances in Childhood Neglect

#### Structural vs. Functional Neuroimaging Findings

Recent neuroimaging studies have provided more precise insights into how childhood neglect impacts both the structure and function of key brain regions involved in executive functioning and emotional regulation, particularly the PFC and the amygdala. For instance, Kawata et al. demonstrated that children exposed to neglect—without co-occurring forms of maltreatment—exhibited significantly reduced gray matter volume in both the PFC and amygdala, as well as diminished functional connectivity between these regions [56]. These neural alterations were strongly associated with inattention and hyperactivity, suggesting a direct neurodevelopmental pathway linking neglect to deficits in behavioral regulation.

To refine this distinction, structural neuroimaging studies consistently report reductions in gray matter volume, cortical thinning, and white matter microstructural disruption, particularly in the hippocampus, amygdala, and PFC. These structural abnormalities have been identified through modalities such as voxel-based morphometry (VBM) [57,58], surface-based morphometry, and diffusion tensor imaging (DTI).

In contrast, functional neuroimaging findings—derived from task-based and resting-state fMRI—reveal hypoactivation of the medial and dorsolateral PFC, as well as disrupted functional connectivity between the amygdala and regulatory prefrontal regions. These alterations are particularly evident during tasks requiring emotion regulation or top-down executive control [59].

Importantly, recent longitudinal studies emphasize that neglect does not affect the PFC globally, but rather targets specific subregions. For example, MRI research has shown significant cortical thinning in the dorsolateral PFC (dlPFC) among children exposed to neglect, while reductions in medial PFC (mPFC) thickness have been linked to impaired emotion regulation and executive dysfunction in adolescents with maltreatment histories [60,61]. Beyond the PFC and limbic structures, neglect has also been associated with alterations in thalamic and striatal regions involved in attention, salience detection, and reward processing.

Additionally, adult resting-state fMRI studies provide insight into the long-term functional sequelae of childhood neglect. For instance, van der Werff et al. found that adults with a history of emotional neglect exhibited reduced resting-state connectivity between the right amygdala and precuneus, as well as between the dorsal anterior cingulate cortex (dACC) and both frontal and parietal cortices, further implicating persistent disruptions in self-referential processing and executive coordination [62].

To enhance clarity and analytic specificity, we now explicitly distinguish structural from functional alterations throughout this section. A summary table (Table 1) has also been included, categorizing findings by neuroimaging modality and measurement type.

Moreover, recent longitudinal evidence has further clarified the neuroanatomical consequences of neglect. Doretto et al. conducted a comparative neuroimaging study examining hippocampal development in children who experienced institutional rearing versus those who suffered family-based neglect. Their findings revealed that while both groups exhibited reduced hippocampal volume compared to non-neglected controls, children reared in institutions showed significantly more pronounced volume loss [66]. This suggests that the type and intensity of neglect—particularly in contexts of extreme social and sensory deprivation—may differentially impact structural brain development. The study also emphasized that early institutional neglect leads to more severe disruptions in hippocampal white and gray matter organization, which are closely tied to deficits in memory consolidation and emotional regulation. These results reinforce the notion that hippocampal vulnerability is highly sensitive to early caregiving environments and highlight the critical role of caregiving context in shaping neurodevelopmental trajectories [66].

Similarly, Hart et al. demonstrated that neglected children showed reduced amygdala volume and decreased cortical thickness in the PFC, which were associated with poor emotion regulation and executive control [61]. Extending this line of evidence, reviewed structural and functional MRI studies confirmed that early neglect results in hypoactivation of the medial and dorsolateral PFC, limiting the brain’s ability to exert top-down control over emotional responses generated by the amygdala [67]. Adding to this, a recent report summarized longitudinal MRI data revealing that children who experienced neglect before 24 months of age exhibited steeper reductions in cortical thickness across adolescence, particularly in the lateral and medial prefrontal areas and the precuneus. These structural changes were not as prominent in children exposed to later neglect, emphasizing the critical role of timing in brain vulnerability [68].

These findings collectively reflect a growing body of evidence emerging from advances in neuroimaging techniques, which have dramatically improved our understanding of how childhood neglect alters brain development. Through functional and structural MRI, researchers have been able to map specific patterns of reduced gray matter volume, altered connectivity, and cortical thinning, particularly within the prefrontal cortex and amygdala—regions critical for executive functioning and emotional regulation. Notably, Herringa et al. found altered amygdala–PFC connectivity in adolescents with early adversity, suggesting compensatory mechanisms during emotional process [59]. Similarly, Bick et al. provided compelling evidence of disrupted connectivity across multiple cortical networks in maltreated children, highlighting the enduring effects of neglect on brain plasticity [58]. These neuroimaging advances do not merely confirm the behavioral consequences of neglect—they provide a mechanistic window into the neurodevelopmental processes that underlie executive function deficits. As the field continues to evolve, imaging biomarkers may play an increasingly critical role in early identification, risk assessment, and targeted intervention for children affected by neglect [61,67].

## 3. Chronic Stress Pathways in Child Neglect

While child abuse and child neglect are often grouped under the broader category of maltreatment, they represent distinct constructs with differing mechanisms and developmental consequences. Child abuse typically involves active harm (e.g., physical or emotional violence), whereas child neglect is characterized by omission, or the failure to meet a child’s basic physical, emotional, or cognitive needs [3,11]. Although both forms of adversity can activate similar stress-related biological pathways—such as the hypothalamic–pituitary–adrenal (HPA) axis and neuroimmune signaling—neglect is uniquely associated with chronic under-stimulation, unpredictability, and the absence of caregiving regulation during critical periods of brain development.

The HPA axis plays a fundamental role in the body’s response to stress or emergency situations. However, this system is particularly vulnerable to early-life experiences such as childhood adversity [69]. The scientific literature provides abundant evidence showing how such experiences can provoke epigenetic changes in two genes directly involved in the stress response. In this regard, studies have demonstrated that adverse experiences during highly sensitive periods—such as the prenatal stage, childhood, or early adolescence—can leave epigenetic marks on genes involved in brain maturation processes. These modifications not only interfere with the child’s neurological development but also contribute to the emergence of behaviors associated with psychopathology [70].

### Epigenetic Modulation of Stress-Response Genes: NR3C1 and FKBP5

NR3C1 is responsible for producing glucocorticoid receptors, which are essential for the stress response. In acute stress situations, it helps stabilize physiological levels through the release of these receptors. However, chronic stress can lead to methylation of this gene in the hippocampus, resulting in a reduction in glucocorticoid receptor production and a subsequent overactivation of the HPA axis [71,72]. Child maltreatment increases methylation of this gene, thus promoting a maladaptive stress response.

Both animal and human studies have demonstrated how maltreatment can alter the methylation of this gene. One of the most significant findings in animal models is presented by Meaney et al., who showed that poor maternal care causes methylation of the gene’s promoter region [73]. Similar findings have been observed in humans in response to various forms of child maltreatment [74,75,76,77,78,79], thus clearly evidencing the link between maltreatment and epigenetic action on the NR3C1 gene.

The FKBP5 gene regulates glucocorticoid activity by inhibiting their binding to receptors. Its overexpression, caused by hypomethylation, results in continuous production. In adults who have experienced maltreatment, lower methylation levels of this gene have been observed, which reduces the activity of glucocorticoid receptors [13,74]. This phenomenon has also been observed in maltreated children or those raised in institutional settings [75,80,81,82]. Furthermore, a negative correlation has been found between maltreatment and FKBP5 methylation [74].

Recent advances in epigenetics have revealed that early-life neglect leaves lasting molecular marks not only on DNA methylation patterns but also across broader regulatory networks, including chromatin remodeling and inflammatory signaling. Foundational work by Weaver et al. demonstrated that variations in maternal care in rodent models lead to altered methylation of the *NR3C1* promoter in the hippocampus, resulting in long-term downregulation of glucocorticoid receptor expression and heightened HPA axis reactivity [12]. This seminal study provided mechanistic support for later findings in humans, including the widely cited study by Klengel et al., which identified allele-specific demethylation of the *FKBP5* gene in individuals exposed to childhood trauma, thereby implicating gene–environment interactions in stress vulnerability [13].

Beyond DNA methylation, early neglect may also alter neuroimmune pathways through epigenetic regulation of pro-inflammatory genes. For example, studies have shown that early-life adversity can induce microglial priming via histone acetylation at TNF-α gene promoters, leading to persistent low-grade neuroinflammation [83,84,85,86]. This process is believed to contribute to disrupted synaptic pruning, aberrant plasticity, and increased emotional reactivity—all of which are tightly linked to executive dysfunction. Taken together, these findings underscore the complex interplay between environmental deprivation and molecular signaling systems that govern neurodevelopmental trajectories [86].

Emerging evidence indicates that neglect and related stressors not only affect DNA methylation but also modulate immune function through histone modifications, particularly acetylation at pro-inflammatory gene promoters [86]. For example, Delpech et al. (2016) demonstrated in rodent models that early maternal separation leads to histone H3 acetylation at the TNF-α promoter in microglial cells, resulting in persistent upregulation of pro-inflammatory cytokines and heightened sensitivity to later stressors [87]. This phenomenon, known as microglial priming, is associated with long-term changes in synaptic plasticity and has been implicated in the pathophysiology of cognitive and affective disorders.

Additionally, Menard et al. Provided evidence that early social stress increases blood–brain barrier permeability, facilitating immune cell infiltration and sustained neuroinflammation in key brain regions such as the prefrontal cortex and hippocampus—structures highly relevant to executive functioning [88]. These studies suggest that epigenetic regulation of neuroimmune responses, particularly through histone acetylation and chromatin remodeling, may constitute a parallel pathway through which early neglect contributes to long-term neurocognitive disruption.

Table 2 provides a detailed summary of key human studies that examine the relationship between childhood adversity and methylation changes in stress-response genes, primarily *NR3C1* and *FKBP5*. For instance, Perroud et al. reported a methylation increase of approximately 5–12% in NR3C1 among adults exposed to severe childhood maltreatment [79], while Klengel et al. found allele-specific demethylation of FKBP5 in trauma-exposed individuals carrying the risk allele, highlighting a clear gene environment interaction [13]. Similarly, Weder et al. and Tyrka et al. identified statistically significant reductions in FKBP5 methylation in maltreated populations, reinforcing the consistency of these epigenetic patterns across studies [81,89].

Incorporating these quantitative findings offers greater granularity and allows for cross-study comparisons regarding the magnitude of epigenetic changes. This is particularly important for understanding the potential biological impact of early adversity on stress regulation systems. Furthermore, by including both single-generation and intergenerational designs (e.g., Ramo-Fernández et al.) the synthesis also captures how these molecular signatures may persist or even propagate across generations [74]. Together with the expanded mechanistic discussion—including neuroimmune signaling and histone acetylation pathways—this table contributes to a more rigorous and integrative understanding of how neglect biologically embeds developmental risk. Such findings broaden the molecular scope of current models, highlighting the convergence of endocrine, epigenetic, and immune mechanisms in shaping developmental outcomes [89].

## 4. The Effect of Child Neglect on EF Development

Numerous scientific studies have documented the consequences of child maltreatment on the development and functioning of executive functions in both children and adults. A commonly reported finding is the clear association between experienced trauma and difficulties in the execution of these higher-order cognitive functions. Child maltreatment, and particularly neglect, has been identified as a significant risk factor that leads to persistent alterations across all dimensions of human development. These effects are not confined to childhood but persist throughout the individual’s lifespan [93]. To clarify, Table 3 reported the relationship between neglect and executive function.

One of the earliest consequences of this form of maltreatment is dysfunction in attachment formation, which is crucial for the proper development of children [94]. Studies have shown that between 60–80% of children who are victims of neglect develop insecure attachment styles [95], negatively affecting their overall development. Research has documented that the type of attachment formed by children, along with parental educational styles, directly influences the development of executive functions. For example, a democratic parenting style is associated with optimal development of executive functions, whereas a permissive style, more commonly observed in neglect cases, contributes to impairments in skills such as cognitive flexibility [96]. Similarly, a clear relationship has been observed between executive function deficits and victims of physical neglect, especially in areas such as mental flexibility, goal setting, attentional control, emotional regulation, and inhibitory control [97,98], as well as difficulties in emotional processing [99].

Calderón and Barrera found lower performance in attentional and memory tasks among maltreatment victims [100]. In 2013, Nikulina and Widom observed that children with histories of sexual abuse and neglect experienced long-term deficits in executive functions, particularly in non-verbal reasoning [101]. Later research by Kim et al. confirmed that these children scored lower in perceptual reasoning, processing speed, psychomotor speed, and working memory [102]. These findings on working memory had also been reported previously [103,104].

In addition to the aforementioned deficits, children who have experienced maltreatment also exhibit difficulties in sustained attention, abstraction processes, and inhibition [102,103], as well as in planning abilities [105]. Furthermore, studies have documented a cognitive delay of up to 25% compared to their chronological age in children who are victims of neglect, affecting both psychological development and various areas of behavior [106,107]. These data are supported by Bengwasan who found that maltreated children generally exhibit lower IQ scores [108]. These results are maintained even after controlling for the possible influence of internalizing disorders such as depression or anxiety [109].

An interesting aspect in this context is the role highlighted by Fares-Otero et al. [110] regarding cognitive reserve. These authors emphasize that cognition, psychosocial functioning, and child maltreatment are moderated by cognitive reserve, which has a significantly positive impact on overall performance.

### Methodological Considerations: Socioeconomic Confounders and Neglect Subtypes Affecting Executive Functions

It is essential to acknowledge that child neglect rarely occurs in isolation, often emerging alongside broader socioeconomic adversity. Many of the studies reviewed were conducted within low-SES populations, where environmental stressors such as poverty, parental mental health issues, housing instability, and reduced access to education may independently contribute to altered neurodevelopment and executive dysfunction. Although some studies attempted to control for SES or used matched samples, not all controlled statistically for SES as a covariate, limiting the precision of causal inferences [57,64]. Future research should systematically incorporate SES as a covariate or utilize designs (e.g., sibling comparisons, propensity matching) that reduce this confounding influence.

Moreover, the literature often fails to differentiate between subtypes of neglect, despite evidence suggesting that physical neglect (e.g., nutritional or sensorimotor deprivation) and emotional neglect (e.g., lack of caregiver responsiveness or stimulation) may lead to distinct neurocognitive outcomes. For example, physical neglect may disproportionately affect somatosensory integration and memory systems, while emotional neglect more strongly impacts emotion regulation and prefrontal–limbic circuitry [111]. While subgroup analyses were limited in many primary studies, this review highlights the need for future investigations to disaggregate neglect subtypes and assess their unique neurodevelopmental signatures, especially in relation to the timing and duration of exposure.

Despite these distinctions, many neurodevelopmental studies fail to disaggregate these subtypes or rely on composite scores of “neglect,” which may obscure important nuances. A clearer differentiation between neglect modalities is crucial for advancing precision in both research and intervention. Future studies should employ designs that allow for subtype-specific analyses, accounting for timing, severity, and duration of each form of neglect.

## 5. Executive Function as a Mediator of Long-Term Risk

During early infancy, children depend on caregivers to manage their bodily states and actions. In nurturing settings, parents help soothe stress and guide emotional and behavioral responses. This co-regulation process gradually enables children to develop self-control, equipping them for academic and social demands at school. When such caregiver support is lacking, children are more exposed to environmental stress. Without someone to help them cope, intense or prolonged stress may overwhelm their capacity to adapt, increasing the risk of developmental difficulties [112]. Thus, children who have experienced neglect often display impairments in core EF domains such as working memory, inhibitory control, and cognitive flexibility. Consequently, EF difficulties may increase the risk for school failure, substance abuse, mental health disorders, and poor social integration in adolescence and adulthood [113,114].

### The Developmental Cascade Model

Several theoretical models help explain the role of EF in developmental pathways affected by early adversity. The Developmental Cascade Model posits that disruptions in early cognitive and emotional regulation can initiate a chain of negative effects across multiple domains of functioning, with EF deficits acting as a central link in this sequence [115,116]. Concretely, this model suggests that early experiences, whether positive or negative, can have cumulative effects across multiple areas of development over time. Difficulties in one domain may lead to problems in others, such as academic performance or peer relationships. These interconnected effects create a chain reaction, shaping long-term developmental outcomes [117].

One of the studies that provides evidence that these developmental outcomes are showed is Scheier et al.’s study, which demonstrated that early neglect impairs executive function development, which mediates long-term risks such as academic failure and mental health issues [118]. Their findings support the Developmental Cascade Model and Toxic Stress Frameworks, highlighting the critical role of EF in shaping developmental trajectories. This underscores the importance of early interventions targeting EF to mitigate adverse outcomes [118]. Additionally, Murray et al., exposed that aggression in early adolescence can lead to internalizing problems through developmental cascades, with peer and teacher relationships playing a mediating role. Their findings highlight the importance of supportive social environments in mitigating the progression from externalizing to internalizing issues [119].

Recent longitudinal studies have highlighted how early life stressors can initiate developmental cascades leading to adverse outcomes in adolescence and adulthood. For instance, Lee et al. found that early contextual risks and negative parenting practices contribute to internalizing symptoms, such as depression and anxiety, in emerging adulthood [120]. Similarly, Otten et al. demonstrated that early childhood stress and poor parent-child interactions are linked to early adolescent substance use, underscoring the importance of early interventions to disrupt these negative developmental trajectories [121]. These findings reinforce the value of the Developmental Cascade Model, emphasizing the need for early, multi-level interventions to alter maladaptive developmental pathways.

Furthermore, recent research has refined our understanding of how executive function serves not merely as a mediator but also as a moderator of risk trajectories. Blair emphasized that EF skills are malleable and highly sensitive to early educational and environmental contexts, suggesting that enhancing EF may buffer against the negative cascade initiated by early adversity [122]. In this light, interventions focused on improving EF in preschool and early school years—such as curricula that promote cognitive self-regulation, emotional awareness, and goal-directed behavior—have demonstrated long-term benefits on academic achievement and social competence.

Another key area of concern is how EF deficits linked to early neglect can contribute to cumulative disadvantage over time. Children with impaired inhibitory control and cognitive flexibility often struggle with adapting to structured environments like school, which can further erode their motivation and self-concept. These challenges may elicit negative responses from educators and peers, reinforcing cycles of exclusion and underachievement. As this feedback loop persists, it can solidify into a developmental trajectory marked by chronic disengagement, school dropout, and socioemotional difficulties [123].

The developmental cascade also intersects with biological mechanisms. Neuroimaging studies show that early life neglect is associated with alterations in prefrontal cortex development, a brain region critical for EF [57]. These structural and functional changes not only explain behavioral manifestations of EF impairment but also represent neurobiological pathways through which early adversity exerts long-term effects on cognition and emotion regulation [57].

**Figure 1:**
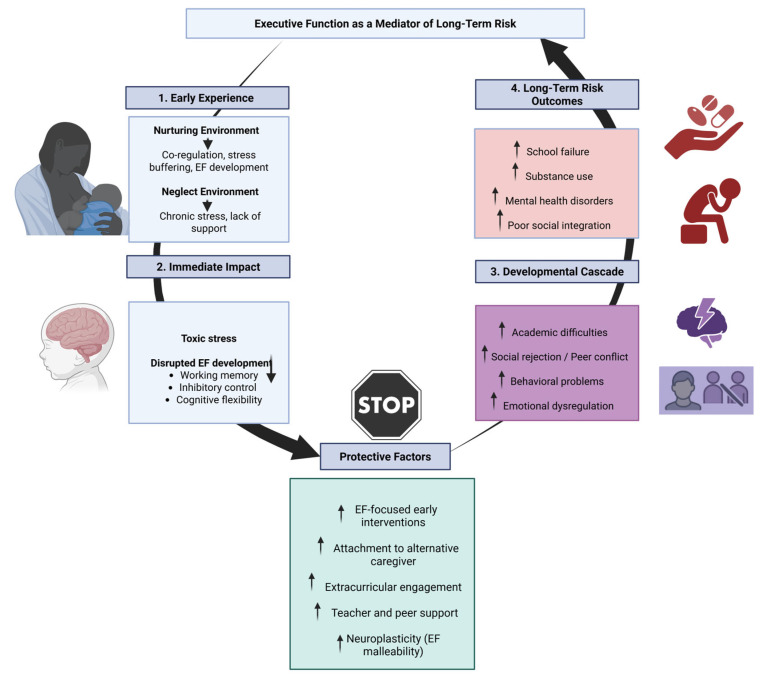
Executive function as a mediator and moderator in developmental cascades after early neglect.

Finally, evidence from resilience research adds a nuanced perspective to the cascade framework. While EF deficits are common among children exposed to neglect, some individuals demonstrate adaptive outcomes despite early risk. Factors such as strong attachment to an alternative caregiver, engagement in extracurricular activities, or involvement in structured mentorship programs have been shown to enhance EF and promote positive developmental outcomes [124]. These findings suggest that protective mechanisms can redirect developmental cascades toward healthier trajectories, reinforcing the importance of timely and context-sensitive interventions (Figure 1).

Taken together, these insights underscore the complex interplay between early experiences, executive functioning, and long-term developmental outcomes. They also affirm the pivotal role of EF as both a risk amplifier and a target for preventive strategies aimed at disrupting maladaptive cascades and fostering resilience.

## 6. Sex Differences in EF Outcomes Following Neglect

Sex-based biological and developmental differences may play a critical role in shaping how early-life neglect affects EF outcomes. Notably, the maturation trajectories of the prefrontal cortex and limbic system differ significantly between males and females. Research suggests that females generally experience earlier prefrontal cortical development, while males exhibit prolonged maturation [125], potentially increasing their vulnerability to disruptions caused by neglect during sensitive periods. In contrast, the limbic system, particularly structures like the amygdala, may develop more rapidly in males, which could heighten emotional reactivity and influence how stress or neglect is processed neurologically [126]. These developmental variations may contribute to sex-specific patterns in EF impairments, such as greater impulsivity or attention difficulties in boys and heightened internalizing symptoms in girls [127]. For instance, Ibrahim et al. found that boys with disruptive behavior exhibited reduced gray matter volume in the left ventromedial prefrontal cortex and decreased cortical thickness in the left supramarginal gyrus, regions associated with emotion regulation and executive functioning. These structural differences were not observed in girls with similar behavioral profiles, suggesting sex-specific neuroanatomical correlates of behavioral dysregulation [126].

In addition, a systematic review by Gaillard et al. examined sex differences in executive control through functional neuroimaging studies. The review found that while both sexes engage similar brain regions during executive tasks, there are notable differences in activation patterns. For instance, females often exhibit greater activation in the prefrontal cortex during tasks requiring cognitive control, suggesting potential differences in how executive functions are processed neurologically between sexes [128,129]. Similarly, Grissom and Reyes highlighted that sex differences in executive function may be influenced by developmental programming and hormonal factors. They emphasized that early-life experiences, including neglect, can differentially impact the development of executive functions in males and females, potentially leading to variations in vulnerability and resilience [104].

### 6.1. Neurohormonal Influences

Sex differences in stress responses and neurodevelopmental outcomes following neglect are significantly shaped by neurohormonal factors, particularly the influence of sex hormones such as estrogen and testosterone, and the differential regulation of the HPA axis [129,130]. Estrogen has been shown to exert neuroprotective effects and modulate stress reactivity, potentially enhancing resilience in females during certain developmental periods [131]. In contrast, testosterone may amplify stress sensitivity and risk-taking behavior, which can influence the trajectory of EF development in males [132]. Additionally, research indicates that females generally exhibit a more reactive HPA axis response to psychosocial stressors, leading to heightened cortisol release and increased vulnerability to internalizing symptoms such as anxiety or depression [133]. Males, on the other hand, often display blunted or delayed HPA responses, which may be linked to externalizing behaviors and impulsivity. For instance, the study by Daughters et al. among adolescents aged 14–18 revealed how psychological stress and cortisol reactivity relate to risk-taking behavior may differ. It is shown that adolescent boys exhibited increased risk-taking behavior following stress exposure, particularly those with a blunted cortisol response and, in contrast, girls demonstrated a decrease in risk-taking behavior post-stress, and their cortisol responses were not significantly associated with changes in risk-taking. These results suggest that in boys, a diminished cortisol response to stress may underlie heightened risk-taking behaviors, whereas in girls, other mechanisms may modulate the relationship between stress and risk-taking [133]. Additional evidence from animal studies supports the idea that early neglect alters neuroendocrine function in a sex-dependent manner. For example, neonatal maternal separation has been shown to differentially affect corticosterone levels and prefrontal cortex development in male and female rodents, with males displaying greater impairments in attentional control and females more susceptible to anxiety-like behaviors [134].These sex-specific neuroendocrine patterns help explain divergent EF outcomes after neglect, with females more prone to deficits in emotional regulation and cognitive control under stress, and males more likely to exhibit attention and behavioral regulation difficulties [135].

In summary, and taken together, neurohormonal and HPA-axis differences help explain sex-specific vulnerabilities in EF development following neglect [136]. Females tend to exhibit heightened emotional reactivity and sustained cortisol responses to stress, which can compromise emotional regulation and cognitive performance under prolonged adversity [137]. These physiological patterns contribute to increased susceptibility to internalizing symptoms, such as anxiety, depression, and rumination, particularly in girls exposed to early adversity [138]. The combination of heightened stress reactivity and maladaptive emotion regulation strategies can also result in perfectionism and cognitive rigidity, which may further impair executive flexibility. In contrast, males more frequently display a blunted cortisol response to stress, a pattern associated with under-arousal, poor impulse control, and increased risk-taking behavior [133,139]. Such hypoactivity in stress-response systems has been linked to deficits in inhibitory control, planning, and behavioral regulation, contributing to externalizing symptoms like aggression, defiance, and conduct problems [140]. Evidence from both human and animal studies further supports that males exposed to early neglect often show impaired attentional control and impulsivity, while females present more anxiety-like behaviors and difficulties in sustained attention and working memory [141,142]. These sex-specific developmental trajectories underscore the need for gender-sensitive approaches to assessment and intervention in children affected by neglect, particularly those targeting executive function and emotion regulation systems.

### 6.2. Women in Contextual Disadvantage: Executive Functions Disruption

As mentioned, recent findings indicate that ACEs, including neglect, have sex-differentiated effects on neurodevelopmental and behavioral outcomes. Women appear to be more adversely affected by neglect than men, likely due to contextual and sociocultural factors that heighten their vulnerability. Cultural norms, gendered expectations, and differential exposure to certain forms of adversity may contribute to a disproportionate impact of neglect on females [143]. Global data show that one in three women has experienced such violence, and women make up most sexual violence victims [144]. Studies also report higher rates of emotional and sexual abuse among women in clinical settings compared to men, highlighting their heightened vulnerability to maltreatment [145]. Additionally, Stoltenborgh and collaborators reported that children of both sexes experience maltreatment, but girls are more often victims of sexual abuse, while boys may face more physical abuse or neglect and are more prone to externalizing outcomes, reflecting sex-specific patterns in both exposure and neurodevelopmental impact [146].

These sex-specific trends in childhood adversity and exposure to different types of maltreatment between genders help contextualize the more severe cognitive and emotional consequences often observed in women, particularly in relation to executive function impairments. Evidence, such as Leza et al.’s research, reported that women undergoing treatment for substance use disorders exhibited higher rates of emotional abuse, sexual abuse, and family mental illness compared to men, and those with three or more ACEs presented more severe addiction-related symptoms and psychopathology [145]. Similarly, outcomes found that emotional abuse and neglect were significantly associated with increased symptom severity, suicide risk, and treatment resistance in patients with obsessive-compulsive disorder, with these effects being more pronounced in women [147,148]. Also, in a study of adolescent girls, Niu et al. identified specific maltreatment profiles linked to increased sexual risk behaviors, underscoring the heightened sensitivity of females to certain ACE-related outcomes [149]. However, Becerra-García et al. found that domestic violence offenders showed impaired executive functioning—specifically reduced cognitive flexibility—compared to non-offenders, suggesting a link between executive deficits and violent behavior [150].

Nevertheless, meta-analytic findings indicate that maltreated girls are more likely to develop disorganized attachment styles, which are closely linked to dysregulated emotional responses and impaired cognitive control [151]. These dynamics are particularly evident in women with histories of complex trauma, who tend to show greater disruption in planning, inhibitory control, and decision-making. Sociocultural expectations may further exacerbate these effects by shaping coping strategies that increase internalization and limit assertive emotional expression [152]. Suchman and collaborators highlighted that, especially those with co-occurring post-traumatic stress and substance use disorders, these deficits are often compounded, manifesting as poor behavioral regulation and difficulty in emotion-guided decision-making. Interventions focused on relational stability, have shown promise in improving executive functions and self-regulation among substance-using mothers [153].

Finally, these findings indicate that executive dysfunction can be present both in victims of maltreatment and in perpetrators of violence, highlighting the complex, bidirectional relationship between adversity, cognitive development, and behavior. These results collectively emphasize the importance of accounting for sex-based differences in the assessment and intervention of executive function impairments following women neglect and adversity, as females may exhibit distinct patterns of emotional, behavioral, and cognitive vulnerability compared to males. For instance, Milligan et al. identified key therapeutic relationship factors, such as empathy, consistency, and a strengths-based approach, that support emotional regulation and executive function among pregnant and parenting women in substance use treatment. Findings highlight the importance of relational dynamics in enhancing cognitive and emotional outcomes within integrated care settings [154].

## 7. Intervention Studies Targeting EF in Neglected Children

Given the well-established impact of early neglect on EF development, a growing body of research has focused on designing and evaluating interventions aimed at strengthening EF skills in affected children. These programs target core domains such as inhibitory control, cognitive flexibility, and working memory, with the goal of improving self-regulation, academic performance, and long-term psychosocial outcomes. Notably, interventions such as *Tools of the Mind*, *PATHS* (Promoting Alternative Thinking Strategies), and various forms of computerized cognitive training have shown promise in enhancing EF capacities [155,156,157]. Moreover, recent advances in developmental neuroscience highlight the potential of such programs to induce measurable changes in brain structure and function, suggesting that EF is a modifiable system responsive to targeted enrichment [157]. Table 1 shows some successful intervention programs for neglected children and the importance of multi-level approaches that integrate family, school, and community contexts. It also addresses the ongoing challenges in ensuring accessibility, cultural relevance, and timely implementation—factors that critically shape the success and sustainability of EF-based interventions in neglected populations.

### 7.1. Implications for Intervention

One of the first steps in the process of interventions is understanding EF as a mediator between early neglect and long-term adverse outcomes has significant implications for intervention and prevention strategies [158]. Then, enhancing EF may serve as a powerful buffer against these negative developmental trajectories. Interventions that target EF—such as programs focused on working memory, inhibitory control, cognitive flexibility, and emotion regulation (Table 4)—can help strengthen children’s capacity for self-regulation and adaptive functioning, even in the context of early life stress [159].

### 7.2. Cultural Feasibility and Global Applicability of Interventions

While promising, most of the interventions reviewed were conducted in high-resource Western contexts, often within well-funded institutional or academic settings [166,169]. This raises important concerns regarding feasibility, scalability, and cultural appropriateness in low- and middle-income countries (LMICs), where child neglect is also highly prevalent but where access to specialized services is often limited [168]. Intervention strategies that rely heavily on trained clinicians, digital platforms, or parent-mediated therapies may face barriers such as caregiver illiteracy, lack of mental health infrastructure, or differing cultural norms around caregiving and child development. Moreover, none of the reviewed studies reported formal assessments of cultural adaptation, which is critical for effective implementation in diverse populations. Future research should prioritize the validation and contextualization of evidence-based programs in LMICs, using community-based participatory designs and evaluating local resources, beliefs, and logistical constraints.

Thus, evidence-based approaches like cognitive training, mindfulness-based practices, and enriched educational environments have shown promise in improving EF skills, particularly when implemented early in development. Moreover, supporting caregivers in promoting co-regulation and providing stable, responsive environments can further reinforce EF development. By focusing on this modifiable domain, interventions can potentially disrupt the cascade of risk initiated by neglect and foster more positive long-term outcomes across cognitive, emotional, and social domains.

## 8. Practical Applications and Limitations

Understanding the neurodevelopmental impact of childhood neglect has direct implications for educational, clinical, and policy-related practices. Early detection protocols in schools and healthcare settings should be refined to identify executive function deficits potentially rooted in neglect. Interventions should prioritize cognitive training programs that target working memory, inhibitory control, and cognitive flexibility, particularly during sensitive developmental periods. Moreover, integrating trauma-informed approaches in educational and therapeutic contexts can foster a supportive environment for affected children, improving academic outcomes and emotional regulation. At a policy level, investing in early childhood programs that ensure caregiver responsiveness and environmental stimulation can act as preventive strategies to reduce the cognitive burden of neglect. In summary:

- Implementation of early screening programs in schools and primary care settings to detect executive function deficits linked to childhood neglect.
- Training educators and mental health professionals in trauma-informed practices to support children with neglect-related neurodevelopmental challenges.
- Development of neuroscience-based cognitive training interventions aimed at enhancing working memory, inhibitory control, and cognitive flexibility.
- Promotion of positive parenting programs and early stimulation strategies, particularly in high-risk populations, to prevent neglect-related developmental impairments.

A key limitation of this narrative review lies in the heterogeneity of the included studies, particularly regarding sample composition and the classification of maltreatment types. In many cases, neglect was not isolated as a distinct exposure but was instead examined alongside other adverse experiences such as abuse or household dysfunction. This overlap makes it difficult to determine whether the observed neurodevelopmental outcomes can be attributed specifically to neglect or to broader patterns of maltreatment. Furthermore, several studies failed to clearly differentiate between subtypes of neglect—for instance, between physical neglect (e.g., nutritional and sensorimotor deprivation) and emotional neglect (e.g., lack of caregiver responsiveness). This lack of specificity limits the ability to draw conclusions about the unique effects of different forms of neglect on brain development. Additionally, while diverse sources were included in the search process, the possibility of publication bias cannot be entirely excluded, as studies with null or inconclusive findings may be underrepresented. Collectively, these limitations highlight the need for future research to employ precise operational definitions, subtype-specific analyses, and consistent control of confounding variables—such as socioeconomic status—to improve the interpretive clarity and scientific rigor of studies on neglect and neurodevelopment. Lastly, the possibility of publication bias must be acknowledged. Because narrative reviews rely on published literature, studies with null or inconclusive findings may be underrepresented. Although we aimed to include a comprehensive range of evidence, this limitation may have influenced the observed patterns in the literature.

## 9. Conclusions

Childhood neglect constitutes a major threat to neurocognitive development, distinct from other forms of maltreatment due to its chronic, under-stimulating nature. The evidence reviewed underscores how neglect disrupts brain systems critical for executive functioning, particularly through alterations in the prefrontal cortex, amygdala, and stress-response mechanisms. Epigenetic findings further reveal how these effects can persist across development, potentially shaping lifelong cognitive and emotional trajectories. Recognizing the unique neurobiological profile of neglect is essential for guiding research, prevention, and intervention strategies aimed at mitigating its long-term impact on mental health and cognitive functioning.

## Figures and Tables

**Table 1:** Synthesis of neuroimaging studies.

Study	Modality	Finding	Region
Adamson et al. (2013) [63]	Structural (VBM)	Reduced GM volume in PFC and hippocampus in neglected children	Prefrontal cortex, hippocampus
McLaughlin et al. (2014) [64]	Structural (Cortical Thickness)	Thinner cortex in mPFC and superior frontal cortex in previously institutionalized youth	mPFC, SFC
Bick et al. (2015) [65]	Structural (DTI)	Reduced white matter integrity in neglected children	Corpus callosum, internal capsule
Silvers et al. (2016) [60]	Functional (fMRI—Emotion Regulation Task)	Altered PFC–amygdala connectivity in maltreated adolescents	PFC, amygdala
van der Werff et al. (2013) [62]	Functional (Resting-state fMRI)	Reduced amygdala–precuneus and dACC–frontal/parietal connectivity in adults with emotional maltreatment history	Amygdala, dACC, precuneus, frontal/parietal cortex
Doretto et al. (2024) [14]	Structural (MRI)	Reduced hippocampal volume in neglected children; more pronounced in institutionalized settings	Hippocampus (gray and white matter)
Hart et al. (2018) [61]	Structural (MRI)	Reduced amygdala volume and cortical thickness in PFC linked to poor emotion regulation	Amygdala, PFC
Herringa et al. (2013) [59]	Functional (fMRI)	Altered amygdala–PFC connectivity in adolescents with early adversity	Amygdala, PFC

**Table 2:** Summary of relevant research in the field.

Authors	Research Results	Gene Under Study
Perroud et al. (2011) [79]	Increased methylation of glucocorticoid receptor gene (NR3C1) in adults with a history of childhood maltreatment: A link with the severity and type of trauma.	NR3C1
Tyrka et al. (2012) [90]	Childhood Adversity and Epigenetic Modulation of the Leukocyte Glucocorticoid Receptor: Preliminary Findings in Healthy Adults.	NR3C1
Klengel et al. (2013) [13]	Allele-specific FKBP5 DNA demethylation mediates gene–childhood trauma interactions.	FKBP5
Weder et al. (2014) [81]	Child abuse, depression, and methylation in genes involved with stress, neural plasticity, and brain circuitry.	FKBP5
Romens et al. (2015) [76]	Associations Between Early Life Stress and Gene Methylation in Children.	NR3C1
Tyrka et al. (2015) [91]	Childhood maltreatment and methylation of FK506 binding protein 5 gene (FKBP5).	FKBP5
Bustamante et al. (2016) [78]	Glucocorticoid receptor DNA methylation, childhood maltreatment and major depression.	NR3C1
Koning et al. (2024) [92]	DNA methylation at stress-related genes is associated with exposure to early life institutionalization.	FKBP5
Cicchetti and Handley (2017) [77]	Methylation of the glucocorticoid receptor gene (NR3C1) in maltreated and nonmaltreated children: Associations with behavioral and emotional symptoms.	NR3C1
Tozzi et al. (2018) [80]	Epigenetic Changes of FKBP5 as a Link Connecting Genetic and Environmental Risk Factors with Structural and Functional Brain Changes in Major Depression.	FKBP5
Ramo-Fernández et al. (2019) [74]	The effects of childhood maltreatment on epigenetic regulation of stress-response associated genes: An intergenerational approach.	NR3C1/FKBP5
Weaver et al. (2004) [12]	Epigenetic programming by maternal behavior affects hippocampal glucocorticoid receptor expression and stress responses.	NR3C1
Delpech et al. (2016) [87]	Early life stress perturbs the maturation of microglia and induces neuroinflammation via histone acetylation at cytokine gene promoters.	TNF/Histone acetylation
Menard et al. (2017) [88]	Stress-induced neuroinflammation alters blood-brain barrier integrity and cognitive function through cytokine pathways.	Neuroimmune pathways

**Table 3:** Relationship between neglect and executive function.

Executive Function	Mediating Mechanism	Observed Impact in Victims of Child Neglect
Working Memory	Lack of cognitive stimulation limits the strengthening of prefrontal and parietal circuits.	Difficulties in retaining and manipulating information.
Planning	The absence of adequate parental models and structured guidance hinders the development of problem-solving schemas.	Problems setting goals, organizing actions, and anticipating consequences.
Inhibition	Chronic stress disrupts limbic system regulation and weakens prefrontal control over emotions.	Low ability to control impulses.
Cognitive Flexibility	Exposure to rigid or overly permissive environments reduces the ability to modify thinking patterns.	Difficulties in shifting strategies or adapting to new rules or contexts.
Sustained Attention	Unstable environments lead to hyperactivation of the alert system and hinder the proper development of attentional networks.	Problems maintaining concentration over time or filtering distractions.
Decision-Making	Lack of adequate adult guidance prevents the internalization of norms and understanding of consequences.	Inappropriate choices across different contexts.
Processing Speed	Prolonged exposure to chronic stress and unstable environments slows neural signal transmission, affecting circuits responsible for cognitive speed.	Delayed information processing and response times.

**Table 4:** Synthesis of interventions.

Author	Title	Results	Intervention	EF Advancements
Arruabarrena et al. [160]	Implementation of an Early Preventive Intervention Programme for Child Neglect: SafeC	High levels of parental satisfaction, significant improvements in parenting skills, and significant decreases in child abuse potential, parental stress, and perception of child behavioral problems were found after treatment	Structured home-visiting intervention aimed at families at risk or showing early signs of neglect toward children aged 0 to 5 years	Enhanced self-regulation
Spawton-Rice and Walker (2020) [161]	Do cognitive training applications improve executive function in children with adverse childhood experiences? A pilot study	The results indicated that the CCTA had a significant positive effect on EF	Computerized cognitive training applications (CCTA) on executive function (EF) in children aged 6 to 11	Enhanced working memory
Lawler et al. (2015) [162]	A Randomized-Controlled Trial of Mindfulness and Executive Function Trainings to Promote Self-Regulation in Internationally Adopted Children	Mindfulness and executive function trainings both hold promise for improving aspects of self-regulation in IA children	Mindfulness training (MT) and executive function training	Reduced hyperactivity and attention problems and improved emotion regulation
Lawler et al. (2019) [163]	A Preliminary, Randomized-Controlled Trial of Mindfulness and Game-Based Executive Function Trainings to Promote Self-Regulation in Internationally Adopted Children	MT participants showed significant improvements in delay of gratification EFT participants exhibited enhanced inhibitory control and selective attention	Interactive, game-oriented activities designed to strengthen cognitive control processes	Improved inhibitory control and selective attention
Bernard et al. (2012) [164]	Enhancing attachment organization among maltreated children: results of a randomized clinical trial	Children in the ABC intervention showed significantly lower rates of disorganized attachment (32%) and higher rates of secure attachment (52%) relative to the control intervention (57% and 33%, respectively)	Attachment and Biobehavioral Catch-up (ABC), an intervention targeting nurturing care among parents identified as being at risk for neglecting their young children	Improvements in children’s EF, including better inhibitory control and attention regulation
Pillhofer et al. (2015) [165]	Pilot study of a program delivered within the regular service system in Germany: effect of a short-term attachment-based intervention on maternal sensitivity in mothers at risk for child abuse and neglect	Infants of mothers in the intervention group showed better emotional development	Home visits and video feedback to promote maternal sensitivity, and was implemented by trained staff within the health care and youth welfare systems	Better emotional development
Souza et al. (2024) [166]	A Naturalistic Intervention to Promote Executive Functions in Primary School Children: A Pilot Study	Greater improvement in executive functions for the Experimental Group, including working memory and inhibition. Additionally, parents and teachers, blind to the experimental conditions, reported improvements in some measures of executive functions and behavior	16 sessions of GMT-based training, incorporating metacognitive strategies, mindfulness, psychoeducation, and cognitive exercises	Significant improvements in working memory and inhibitory control.
Mishra et al. (2020) [167]	Closed-loop digital meditation for neurocognitive and behavioral development in adolescents with childhood neglect	Strengthened dACC connectivity within the cingulo-opercular network, enhanced sustained attention and interference resolution, and reduced hyperactivity at one-year follow-up. Adolescents also showed improved academic performance, with all outcomes significantly correlated with changes in dACC connectivity	Closed-loop digital meditation program designed to enhance neurocognitive processes	Improved core executive functions, including sustained attention, interference resolution, and self-regulation. These gains were associated with better academic performance
Roque-Lopez et al. (2021) [168]	Mental health benefits of a 1-week intensive multimodal group program for adolescents with multiple adverse childhood experiences	After completing the program, the intervention group showed significant reduction in trauma-related outcomes while attention/awareness-related outcomes were improved by 57%	The intervention included mindfulness-based practices, expressive arts and EMDR (Eye Movement Desensitization and Reprocessing Integrative) group treatment	Improvements in executive functions, such as enhanced self-regulation, better emotional control, and improved attention
Raver et al. (2011) [169]	CSRP’s Impact on low-income preschoolers’ preacademic skills: self-regulation as a mediating mechanism	Chicago School Readiness Project improved low-income children’s self-regulation skills (as indexed by attention/impulse control and executive function) from fall to spring of the Head Start year	Teacher training and in-class mental health consultation to improve classroom climate and support children’s self-regulation	Improvement in children’s self-regulation
Demeusy et al. (2020) [170]	A Multi-Component Intervention to Prevent Child Maltreatment: Long-term Effects on Parenting and Child Functioning	The Building Healthy Children (BHC) intervention in children exhibited less externalizing behavior and self-regulatory problems at follow-up, across parent and teacher report	BHC flexibly delivers three evidence-based treatment models based on individual need in conjunction with continuous outreach support. These models addressed parenting (Parents as Teachers), attachment (Child-Parent Psychotherapy), and maternal depression (Interpersonal Psychotherapy for Depressed Adolescents)	Enhancements in children’s self-regulation and executive functioning

## Data Availability

Not applicable.
